# A safe foodstuff for wheat allergic patients

**DOI:** 10.1186/2045-7022-4-S2-P59

**Published:** 2014-03-17

**Authors:** María Garrido-Arandia, PM Gamboa, C González, C Pereira, M Catarino, E García-Lirio, A Soriano, LF Pacios, A Díaz-Perales

**Affiliations:** 1 Technical University Madrid, CBGP (UPM-INIA), Pozuelo de Alarcón (Madrid), Spain; 2 University Hospital Basurto, Allergology Service, Bilbao, Spain; 3 University Hospital Basurto, Pediatrics Service, Bilbao, Spain; 4 University Faculty of Pharmacy, Lisbon, Portugal

## Background

Lipid Transfer Protein (LTP) allergy constitutes the first cause of food allergy to vegetables in Southern Europe. LTPs are highly stable proteins, resistant to both gastric digestion and high temperatures. Allergic reactions to multiples vegetables, not phylogenetically related, are produced based on the cross-reactivity among these proteins, a phenomenon that has been named LTP syndrome. Tri a 14, the wheat LTP, is involved in baker´s asthma and anaphylactic reaction. In the present work, we studied Weetabix ® foodstuff tolerance in four patients with wheat allergy, characterized by oral food challenge (OFC) and skin tests, trying to identify the source of this tolerance in wheat-based foodstuff.

## Methods

Four patients with LTP syndrome, showing confirmed Tri a 14-mediated cereal allergy by wheat OFC and positive skin test to wheat extract, nPru p 3 and Tri a 14 were included in the study. Individual sera from these patients were incubated with a homemade allergen array including 12 wheat allergens, Pru p 3 and several pollen allergens. Molecular characterization of Weetabix foodstuff was determined by SDS-PAGE and mass spectrometry analysis.

## Results

All patients showed specific IgE binding to Pru p 3 and Tri a 14 with our arrays. Oral challenge with Weetabix foodstuff was performed in these patients who tolerated it without suffering from any symptoms. Weetabix extract SDS-PAGE and mass-spectrometry analysis show no bands and peaks lower than 2 kDa.

## Conclusions

Pressure and high temperature treatment seemed sufficient to inhibit the allergenic activity of LTPs. This prepared foodstuff could be a safe alternative for these patients with wheat allergy by LTP sensitization and this process could be applied to other plant foodstuffs, in order to achieve LTPs proteolysis.

